# Therapeutic potential of the topical recombinant human interleukin-1 receptor antagonist in guinea pigs with allergic rhinitis

**DOI:** 10.1186/s13223-024-00893-9

**Published:** 2024-06-04

**Authors:** Haibing Li, Chanjuan Di, Yanbing Xie, Yuexia Bai, Yongxue Liu

**Affiliations:** 1 Department of Pharmacy, Yingtan 184 Hospital, Yingtan, 335000 Jiangxi China; 2 Department of Pharmacology and Toxicology, Beijing Institute of Radiation Medicine, No. 27, Taiping Rd., Haidian District, Beijing, 100850 China; 3 Department of Pathology, Qilu Children’s Hospital of Shandong University, Shandong, 250022 China

**Keywords:** rhIL-1Ra, Ovalbumin, Allergic rhinitis, Guinea pig

## Abstract

**Background:**

Recombinant human Interleukin receptor antagonist (rhIL-Ra) can bind to the IL-1 receptor on the cell membrane and reversibly blocks the proinflammatory signaling pathway. However, its effect on allergic rhinitis (AR) and the underlying mechanism remains unknown. This study aims to investigate the efficacy of recombinant human interleukin-1 receptor antagonist (rhIL-1Ra) on AR guinea pigs.

**Methods:**

Guinea pigs were systemically sensitized by intraperitoneal injection and topical intranasal instillation with ovalbumin within 21 days. Animals administrated with saline served as the normal control. The AR animals were randomly divided into the model group and distinct concentrations of rhIL-1Ra and budesonide treatment groups. IL-1β and ovalbumin specific IgE levels were detected by ELISA kits. Nasal mucosa tissues were stained with hematoxylin & eosin (HE) for histological examination.

**Results:**

It was found that the numbers of sneezing and nose rubbing were remarkably reduced in rhIL-1Ra and budesonide-treated guinea pigs. Besides, rhIL-1Ra distinctly alleviated IgE levels in serum and IL-1β levels in nasal mucus, together with decreased exfoliation of epithelial cells, eosinophilic infiltration, tissue edema and vascular dilatation.

**Conclusions:**

rhIL-1Ra is effective in AR guinea pigs and may provide a novel potential choice for AR treatments.

## Background

Allergic rhinitis (AR) is defined as an IgE-mediated inflammatory disease characterized by the symptoms like sneezing, nose rubbing, rhinorrhea, lacrimation and nasal congestion. The global prevalence of AR patients is increasing year by year, and they usually exhibit symptoms such as generalized discomfort, depression, and anxiety, which seriously reduce the quality of life [1]. Currently, although some measures have been taken for the treatment of AR, such as medication and immunotherapy, they also bring some disadvantages to AR treatment to a certain extent. In particular, long-term use of first- and second-generation antihistamines can lead to central nervous system depression and sedative-hypnotic tolerance, and even result in arrhythmias in some AR patients [2, 3]. Some patients treated with intranasal corticosteroids may exist perforated nasal septum ulcers, decreased endogenous cortisol secretion, and adverse symptoms such as nasal bleeding and dry nose [1, 4, 5]. Therefore, the search for new and safer drugs to improve the efficacy of AR remains an urgent issue.

Interleukin-1 (IL-1) functions as one of the well-characterized pro-inflammatory cytokines that has significant biologic activities. As a lymphocyte-activating factor, IL-1 improves B cells activation leading to an increase in IgE production, and enhances mast cell cytokine secretion and histamine release [6], suggesting its close relationship with AR. Interleukin-1 receptor antagonist (IL-1Ra) is a natural and desirable anti-inflammatory cytokine that inhibits cytokine function by binding competitively to IL-1R1. However, studies have shown that endogenous levels of IL-1Ra produced in the synovial fluid of some arthritis patients are insufficient to alleviate the increase in IL-1Rs [7]. Therefore, exogenous administration of IL-1Ra is very important for anti-inflammation. Recombinant human IL-1Ra (rhIL-1Ra) as a genetically engineered drug has been found to have the same biological function as natural IL-1Ra both of which can bind specifically to IL-1R to inhibit various inflammatory diseases, like encephalomyelitis [8], intestinal mucositis [9], arthritis [10], and even cancer [11, 12]. Recently, it has been approved by the US FDA for the clinical treatment of rheumatoid arthritis (RA) [13], which further aroused our interest in its treatment of AR.

In the present study, we investigated the role of rhIL-1Ra in guinea pigs with AR. We demonstrated that rhIL-1Ra remarkably alleviated the symptoms like sneezing and nose rubbing. Moreover, rhIL-1Ra reduced IL-1β and ovalbumin specific IgE levels as well as epithelial cell exfoliation and eosinophilic infiltration. These effects may confer the therapeutic effects of rhIL-1Ra as revealed by in vivo studies.

## Methods

### Animals

Male Hartley guinea pigs, weighing 300–350 g, were purchased from Vital River Laboratory Animal Technology Co. Ltd. Animals were fed and housed in polypropylene cages containing sterile sawdust at the Animal Center, with the controlled condition of automatic 12 h light/dark cycles, 23 ± 2 °C temperature and 55 ± 5% humidity, and were given water and food ad libitum. This animal experiment was approved by the Ethics Committee of Beijing Institute of Radiation Medicine.

### Materials

Ovalbumin (Grade V) and Aluminium hydroxide gel were purchased from Sigma, USA. rhIL-1Ra were supplied by Beijing C&N International Sci-tech Co., Ltd. Budesonide nasal spray was purchased from AstraZeneca Pharmaceutical Limited Company. Guinea Pig Ovalbumin Specific IgE ELISA kit was obtained from Shanghai Tianhong Biotechnology Co., Ltd. ELISA Kit for Interleukin 1 Beta (IL-1β) was purchased from Wuhan USCN Business Co., Ltd. Ovalbumin was dissolved in 0.9% saline at a concentration of 60 mg/ml on use.

### Systemic sensitization and intranasal challenge

To induce AR, the sensitization procedure was performed as described by Underwood and his group [14]. Seventy healthy animals were randomly divided into control (*n* = 10) and model (*n* = 60) groups. On Day 1, 4, 7, 11, 14, 18 and 21, animals were sensitized by intraperitoneal injection with 0.8 mL suspensions containing ovalbumin (200 μg/animal) and aluminium hydroxide gel (0.3 mL/animal), respectively. Ovalbumin suspension with 60 mg/mL was locally dropped once daily in the nostril of the sensitized animals on Day 8, 10, 13, 17 and 20. Animals in the control group received equal volumes of saline. Twenty-eight days later, animals were intradermal injected with 25 μL ovalbumin suspension (200 μg/mL) at the dorsal back surface. Redness and edema at the injection site indicates successful sensitization. Each sensitized guinea pig was given 60 mg ovalbumin suspension (20 μL/400 g) intranasally once daily for a week (from day 29 to day 35).

### Symptoms

Due to the sensitivity and timidity of guinea pigs, the animal room should be quiet and keep the temperature and humidity constant. Five to six animals were placed in an observation cage. Ovalbumin suspension was dropped into both nostrils of the animals, and subsequently clinical symptoms of typical AR of animals in distinct groups, such as sneezing and nose rubbing, were observed for one hour. Sensitized animals usually produce lacrimation and rhinorrhea symptoms, accompanied by sneezing. For lacrimation evaluation, +, ++ and +++ stand for mild (hazy eye), moderate (lacrimation) and severe (lacrimation with noticeable conjunctivitis), respectively [15]. For rhinorrhea evaluation, -, +, ++ and +++ stand for none, mild (within the nasal cavity or spraying on the walls of cage), moderate (outside the nasal cavity) and severe (spilling all the nasal), respectively. For nasal congestion evaluation, -, +, ++ and +++ denote none, mild (impaired inspiration), moderate (nasal crackles) and severe (severe breathing impairment), respectively [16, 17].

### Pharmacodynamic evaluation

After establishment of AR models, forty-six sensitized animals were randomly divided into five groups: the model group (*n* = 10), budesonide treatment group (*n* = 9), and 50 μg/kg, 100 μg/kg, and 200 μg/kg of rhIL-1Ra treatment groups (*n* = 9), respectively. Animals without any treatment were considered as the control group (*n* = 10). Guinea pigs in the treatment groups were administrated with budesonide (25.6 μg/kg, 10 μL/kg), and 50 μg/kg, 100 μg/kg, and 200 μg/kg of rhIL-1Ra (10 μL/kg) for 1 h, respectively, and afterwards ovoalbumin suspension was dropped into both nostrils of the sensitized animals. Guinea pigs in control and model groups were given a certain volume of saline (10 μL/kg). AR symptoms were then assessed within 1 h. Then, animals were treated with saline, budesonide and distinct concentrations of rhIL-1Ra, respectively, once daily for two weeks.

### Measurement of ovalbumin-specific serum IgE level

Ovalbumin-specific IgE levels in guinea pigs were detected using commercial kits according to the manufacturer’s instruction. Briefly, blood samples were collected and centrifuged at 3000 rpm for 10 min at 4 °C at the end of the experiment, and then these samples were stored at -80 °C for later use.

### Measurement of IL-1β in nasal lavage fluid

The secretion of cytokines in nasal mucus is of great significance in investigating the pathological mechanism of AR. In this study, 0.6–0.7 mL of nasal lavage fluid was collected with sterile plastic tubes via dropping 1 mL saline into the animal’s nasal cavity. The nasal lavage fluid was separated by centrifugation (3000 rpm for 10 min at 4 °C). Finally, samples were determined using ELISA kits for Interleukin 1 Beta.

### Histological analysis

After collecting the nasal lavage fluid, guinea pigs were sacrificed for histological examination. Nasal mucosa was separated and fixed in formalin solutions, embedded in paraffin, and stained with HE. Five regions were randomly selected for each section and evaluated by experienced pathologists in a blind way.

### Statistical analysis

All statistical analyses were performed with SPSS 13.0 for Windows. All data are expressed as means ± SEM. One-way ANOVA followed by Dunnett test was employed to estimate the difference between multiple groups. Two-way ANOVA followed by Tukey’s post-hoc test was used to assess symptoms (sneezing and nose rubbing) of animals in multiple groups. *P* < 0.05 was considered as a significant difference.

## Results

### Effect of topical rhIL-1Ra on ovalbumin-mediated symptoms of AR guinea pigs

Previously, we specifically explored the construction of guinea pig AR models, which are induced by a two-step process of sensitization and stimulation [17]. We found that clinical symptoms (duration, severity and frequency of nasal itching, sneezing and runny nose, etc.), histamine contents of nasal mucosa, serum IgE, eosinophilic infiltration of nasal mucosa, small blood vessel dilation, tissue edema and epithelial shedding of animals in the model group were significantly higher than those in the control group [17], implying that the construction of this AR model was successful. This model has been reported in many literatures [1, 18–21]. Based on these findings, we further explored the effect of topical rhIL-1Ra on the symptoms of AR guinea pigs.

**Fig. 1:**
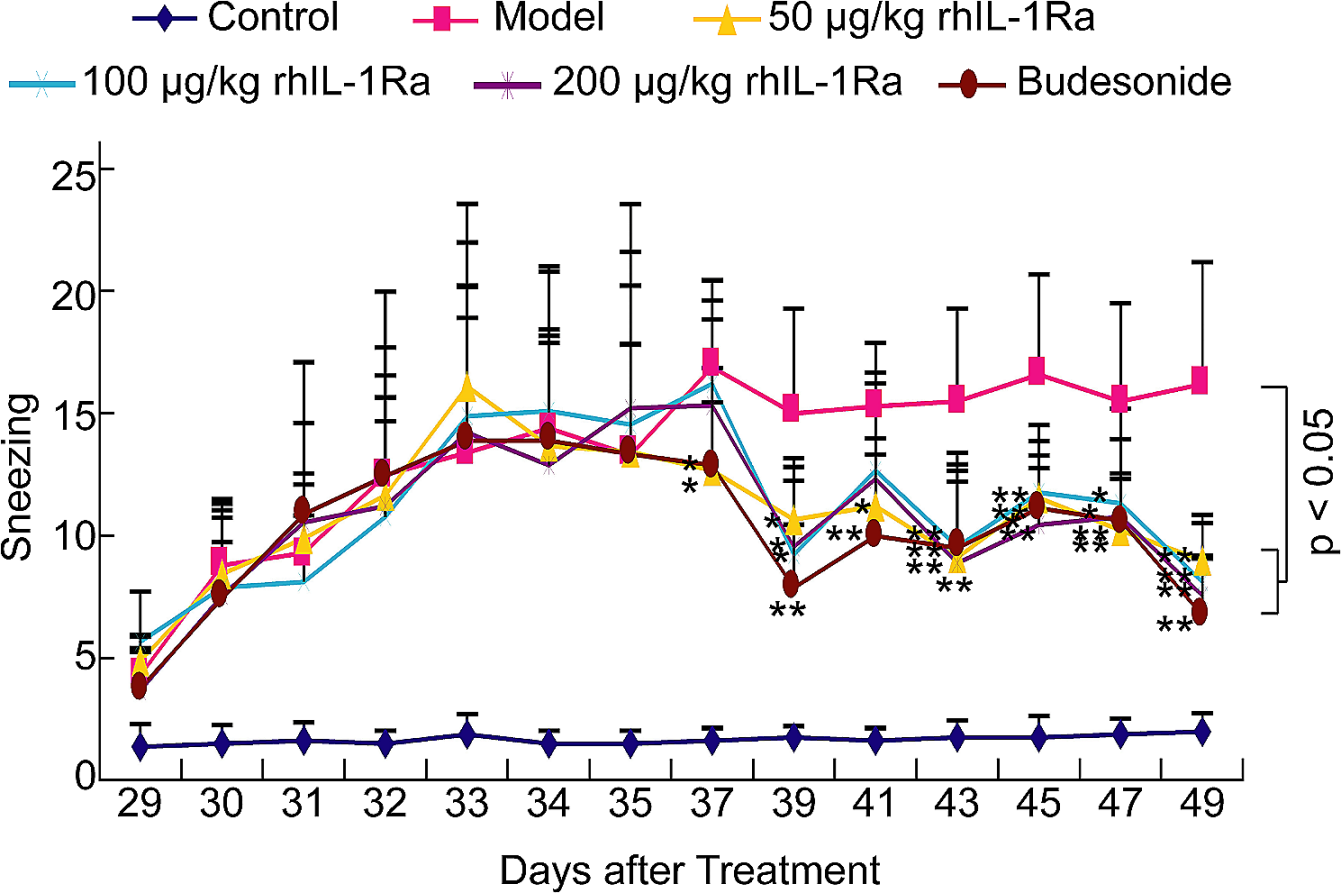
Effects of topical treatment with rhIL-1Ra and budesonide two weeks on the sneezing challenged by ovalbumin in guinea pigs (*n* = 10, means ± SEM). The number of sneezing was sharply decreased in rhIL-1Ra and budesonide treatment groups at Day 49. *P* < 0.05 compared with the Model group

**Fig. 2:**
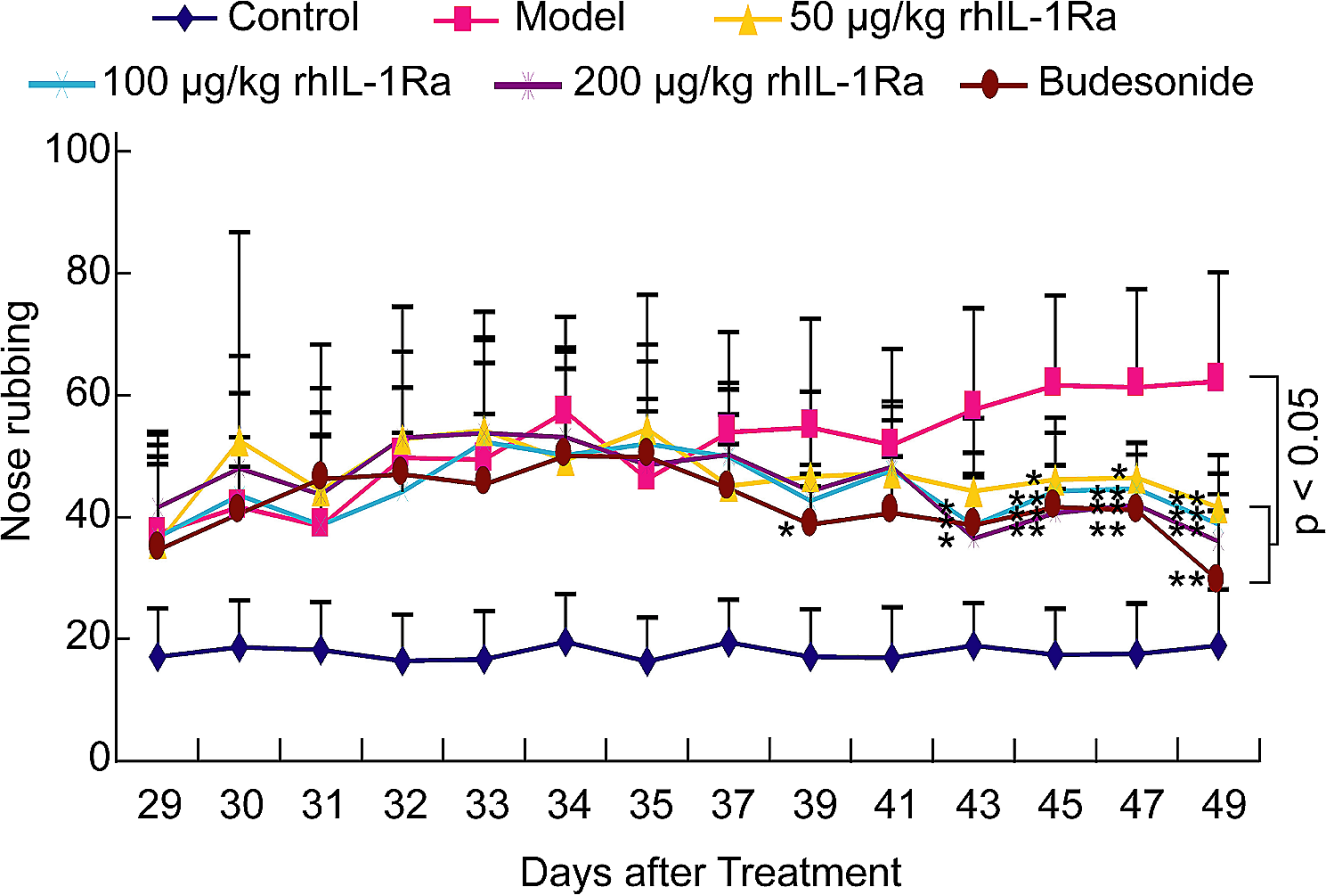
Effects of topical treatment with rhIL-1Ra and budesonide two weeks on nose rubbing challenged by ovalbumin in guinea pigs (*n* = 10, means ± SEM). The number of nose rubbing was sharply reduced in rhIL-1Ra and budesonide treatment groups at Day 49. *P* < 0.05 compared with the Model group

Sneezing and nose rubbing are generally considered to be key indicators for the evaluation of AR in animals [22–24]. As shown in Figs. 1 and 2; Table 1, the numbers of sneezing and nose rubbing of animals in the control group were 2.0 ± 0.8 and 18.9 ± 9.2 on Day 49, respectively, and yet both sneezing (16.2 ± 5.0) and nose rubbing (62.3 ± 17.9) were sharply increased in the model group. Treatment with 50 μg/kg (9.0 ± 1.9), 100 μg/kg (8.1 ± 2.4), and 200 μg/kg (7.6 ± 1.5) of rhIL-1Ra prominently decreased ovalbumin-mediated sneezing comparing to the model group. Besides, the frequency of nose rubbing was also markedly reduced in guinea pigs treated with increasing concentrations of rhIL-1Ra (41.6 ± 8.6, 38.8 ± 8.4, 36.0 ± 7.8). Budesonide was used as a positive drug. It was found that the numbers of sneezing and nose rubbing of animals in budesonide-treated group decreased to 6.8 ± 2.4 and 29.5 ± 11.5 on Day 49, respectively, which is similar to the results in rhIL-1Ra-treated groups. In addition to sneezing and nose rubbing, we also evaluated other typical clinical symptoms of AR on Day 49, such as lacrimation, congestion and rhinorrhea. Our results showed that treatment of distinct concentrations of rhIL-1Ra and budesonide also significantly reduced the severity of lacrimation, congestion and rhinorrhea of guinea pigs mediated by ovalbumin comparing to that of animals in the model group (Table 1). Collectively, these results demonstrated that rhIL-1Ra may have excellent therapeutic effects in guinea pigs with AR.

**Table 1:** Effects of topical rhIL-1Ra on ovalbumin-induced of AR symptoms on Day 49

Group	Number	Frequency/severity of symptoms within 1 h				
		Sneezing	Nose rubbing	Lacrimation	Congestion	Rhinorrhea
Control	10	2.0 ± 0.8	18.9 ± 9.2	+	-	-
Model	10	16.2 ± 5.0^Δ^	62.3 ± 17.9^Δ^	+++	+++	+
50 μg/kg rhIL-Ra	9	9.0 ± 1.9^*^	41.6 ± 8.6^*^	++	+	-
100 μg/kg rhIL-Ra	9	8.1 ± 2.4^*^	38.8 ± 8.4^*^	++	-	-
200 μg/kg rhIL-Ra	9	7.6 ± 1.5^*^	36.0 ± 7.8^*^	+	-	-
Budesonide	9	6.8 ± 2.4^*^	29.5 ± 11.5^*^	+	-	-

#*p* < 0.05, compared with the control group; *p < 0.05, compared with the model group

### Effect of topical rhIL-1Ra on IL-1β levels in nasal lavage fluid

**Fig. 3:**
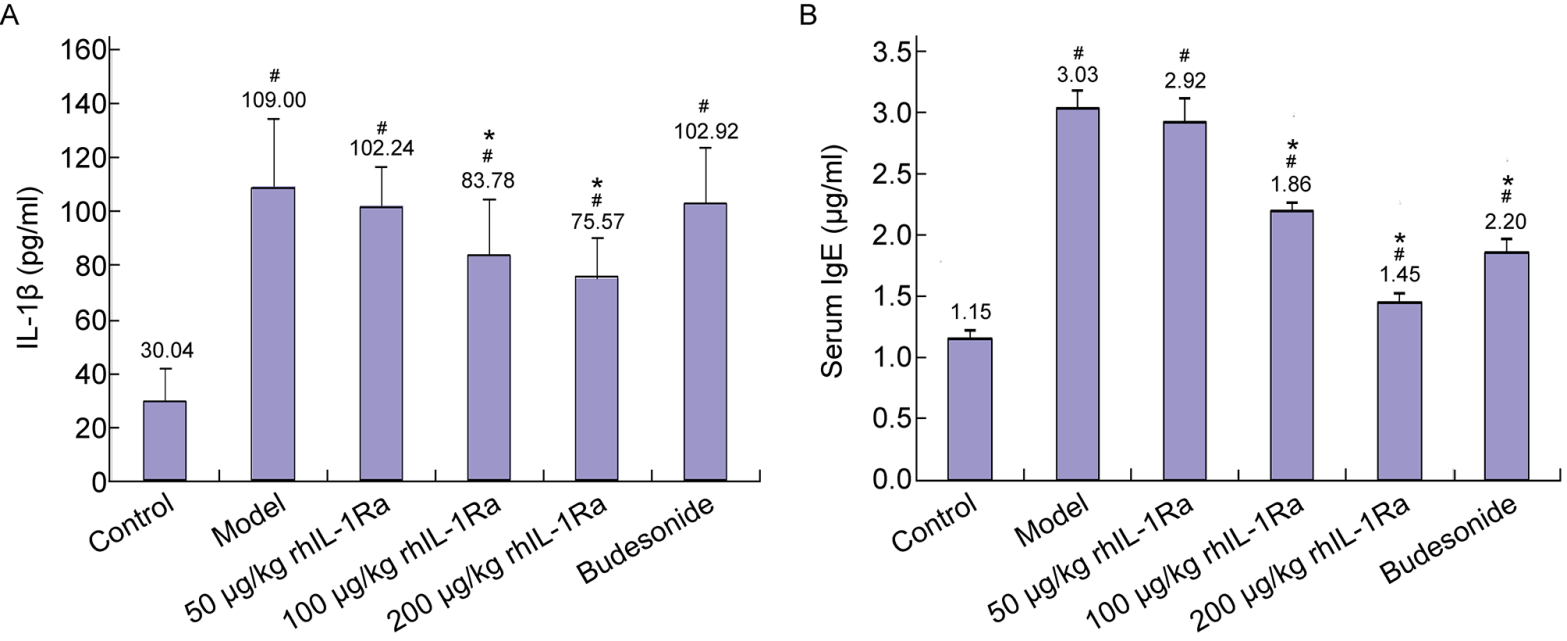
Effects of topical treatment with rhIL-1Ra and budesonide two weeks on the levels of IL-1β in nasal lavage and ovalbumin specific serum IgE in guinea pigs (*n* = 10, means ± SEM). (**A**) Detection of IL-1β levels. (**B**) Detection of ovalbumin specific serum IgE levels. #*P* < 0.05, compared with the control group; *P < 0.05, compared with the Model group

Increasing evidence indicates that IL-1β is a critical molecule in AR and its release contributes to the inflammatory process. To investigate the effect of rhIL-1Ra on IL-1β, nasal lavage fluid of animals in each group was collected to detect IL-1β levels. The results showed that IL-1β levels were markedly elevated in ovalbumin-mediated AR guinea pig models (Fig. 3A). However, treatment with 100 μg/kg and 200 μg/kg of rhIL-1Ra for two weeks notably reduced the increased levels of IL-1β (Fig. 3A). Surprisingly, budesonide treatment did not affect IL-1β levels (Fig. 3A). These results indicated that rhIL-1Ra could decrease IL-1β levels in AR guinea pig models.

### Effect of topical rhIL-1Ra on ovalbumin-specific serum IgE levels

Next, we determined the levels of ovalbumin-specific IgE in serum via the ELISA method. Compared with the control group, animals in model group had markedly increased serum ovalbumin-specific IgE levels (Fig. 3B). However, the IgE levels were reduced in 100 μg/kg and 200 μg/kg of rhIL-1Ra and budesonide treatment groups comparing to guinea pigs in the model group (Fig. 3B). These results suggested that rhIL-1Ra also could decrease ovalbumin-specific serum IgE levels in AR guinea pig models.

### Effect of topical rhIL-1Ra on histological changes

**Fig. 4:**
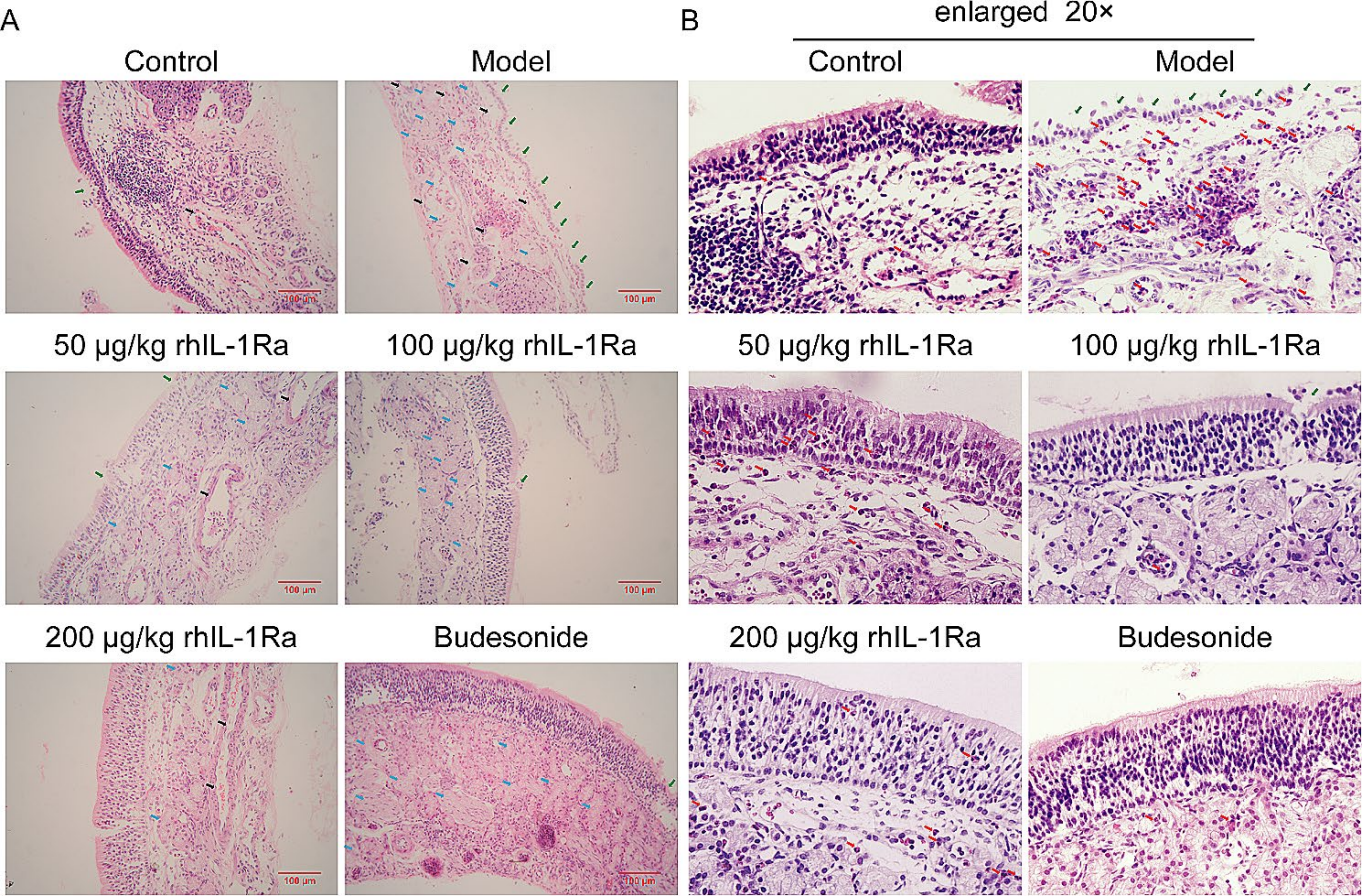
HE staining of nasal mucosa in guinea pigs of the control and rhIL-1Ra and budesonide treatment groups. (**A**) Animals were divided into 6 groups: the healthy control group (control), the AR guinea pig model group (model), distinct concentrations of rhIL-1Ra-treated groups (50 μg/kg, 100 μg/kg, and 200 μg/kg rhIL-1Ra), and the positive drug group (budesonide). Scale bar = 100 μm. (**B**) The HE-stained images of the groups in Fig. 4A were magnified 20 times. Exfoliation of epithelial cells (green arrows) and eosinophilic infiltration (red arrows)

Histopathologic examination of nasal mucosa revealed that epithelial cells of the nasal mucosa were arranged regularly and columnar with none of inflammatory cell infiltration, tissue edema and vasodilation in the control group (Fig. 4A and B), while the epithelial structure of nasal mucosa of guinea pigs in the model group was seriously damaged, which was characterized by severe exfoliation of epithelial cells and eosinophilic infiltration in the lamina propria (Fig. 4A and B). In contrast, treatment with increasing concentrations of rhIL-1Ra and budesonide decreased exfoliation of epithelial cells and alleviated eosinophilic infiltration (Fig. 4A and B). These results indicated that rhIL-1Ra significantly improved the damage of nasal mucosa in AR guinea pig models.

## Discussion

AR is an allergic disease of the nasal mucosa mediated by an inflammatory mediator IgE, which seriously affects people’s quality of life and daily activities. As a type of rhinitis, AR has a prevalence of 30% and is growing rapidly worldwide. It is usually associated with related inducers and specific IgE antibodies; due to its common genetic origin, asthma and eczema are asthma and eczema are frequently found to coexist in adults and children. These findings suggest that effective treatment of AR is a key strategy for asthma prevention [25]. Currently, AR treatment can be divided into the following main categories, including drug therapy, immunotherapy, and surgery. Among them, the use of antihistamines and topical steroids plays positive and crucial roles in improving the patients’ quality of life. Particularly, different dosage formulations of antihistamines (oral liquids, eye and nose drops) are widely used to treat mild to severe AR. However, first and second generation antihistamines have been found to exert therapeutic limitations, such as central nervous system depression and sedative-hypnotic tolerance after long-term use [2]. Some of these drugs also show low specificity and side effects, including thirst, urine retention, tachycardia, etc [26]. . , and are able to cross the blood-brain barrier [3], leading to arrhythmias in some patients with AR, along with anti-α-adrenergic, anti-muscarinic, and anti-serotonin effects [3]. Patients with long-term use of intranasal corticosteroids often lead to septal ulcer perforation and decreased endogenous cortisol secretion [4], accompanied by a number of adverse symptoms, such as nasal dryness, nasal bleeding, and tingling sensation, etc [5]. . . Therefore, it is of interest to investigate safe and effective anti-AR drugs.

Upon exposure to allergens initially, antigen presenting cells (APC) in the body, i.e., macrophages and dendritic cells (DC), present allergens to CD4 + T lymphocytes, the latter of which release distinct cytokines to stimulate B lymphocytes to transform into plasma cells, and to release IgE binding to mast cells and eosinophils, etc [27]. . Mast cells, eosinophils, basophils and lymphocytes play key roles in releasing the pro-inflammatory mediators, including histamine and cytokines, which are all characteristics of the AR response [28–30]. In addition, pro-inflammatory cytokines induce nasal symptoms and aggravate the inflammatory of AR.

Numerous studies have shown that IL-1 serves as a pro-inflammatory cytokine that contributes to B-cell activation and increased IgE production [31, 32]. However, IL-1Ra inhibits cytokine function by competitively binding to IL-1R. Recently, the genetically engineered drug rhIL-1Ra has been found to bind specifically to IL-1R to antagonize various diseases caused by IL-1 overexpression, such as rheumatoid arthritis (RA), sepsis, type II diabetes, and other inflammatory diseases [33]. This further aroused our interest in its treatment of AR.

In a previous study, we successfully constructed AR guinea pig models [17]. Based on this, we further explored the effect of topical rhIL-1Ra on the symptoms of AR. In this study, we found that both sneezing and nose rubbing were sharply elevated on Day 49 in ovalbumin-mediated AR guinea pig models, while treatment with rhIL-1Ra significantly decreased the frequency of sneezing and nose rubbing in guinea pigs (Figs. 1 and 2; Table 1), which is consistent with the results of some published papers [22–24]. In addition, rhIL-1Ra also remarkably alleviated the severity of lacrimation, congestion and rhinorrhea of AR animals (Table 1). Taken together, these data confirmed the excellent therapeutic effects of rhIL-1Ra in guinea pigs with AR.

**Fig. 5:**
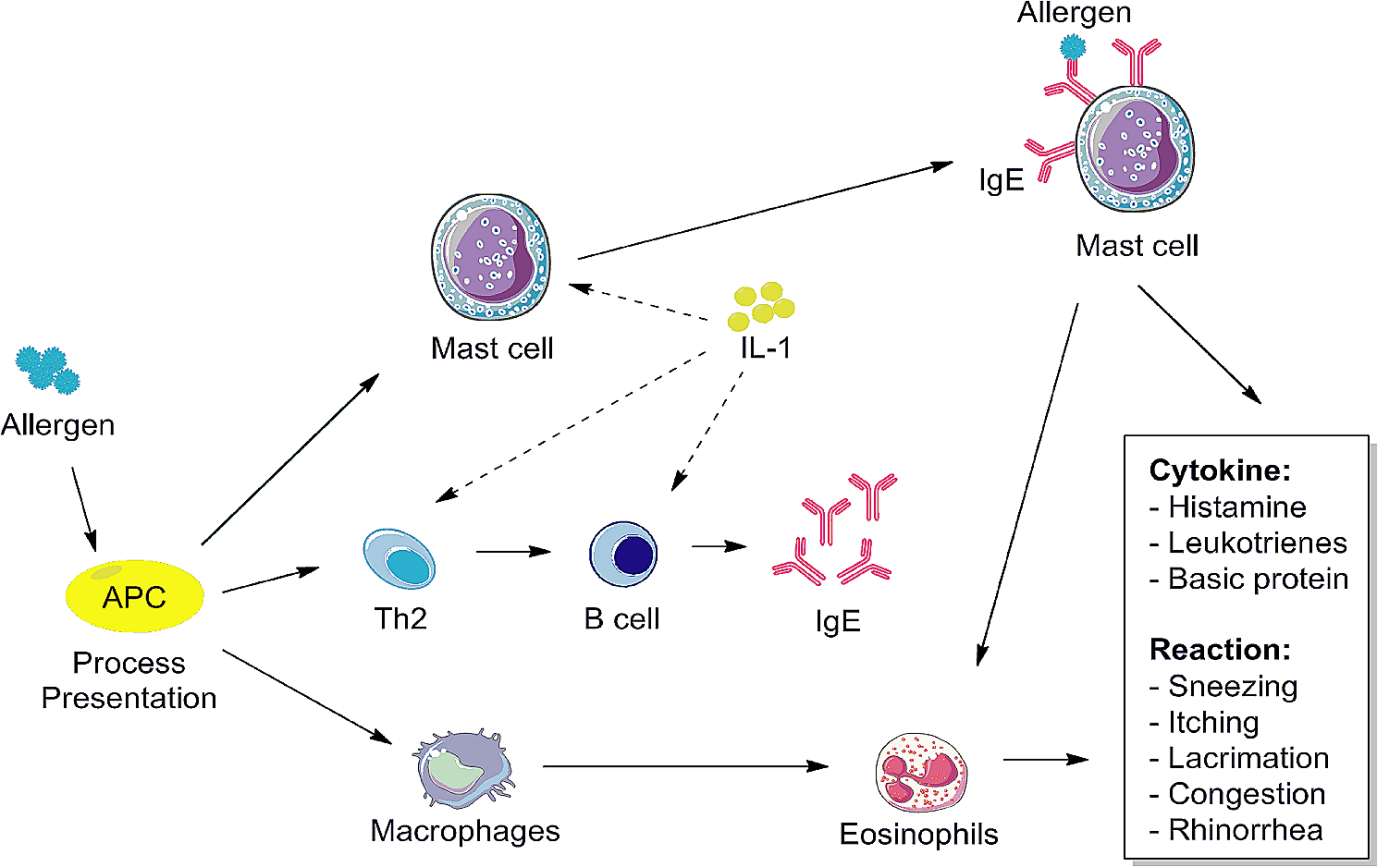
Schematic diagram showing the potential mechanisms for the therapeutic effects of rhIL-1Ra on AR guinea pig models. rhIL-1Ra treatment alleviates rhinitis symptoms, like snnezing, nose rubbing, lacrimation, congestion and rhinorrhea, thorugh reducing IL-1β and ovalbumin specific IgE levels. Meanwhile, rhIL-1Ra causes decreased exfoliation of epithelial cells and eosinophilic infiltration

Allergic inflammation is associated with the imbalance of TH1 vs. TH2 cytokine expression, which leads to over activity of TH2 [34]. TH1 cells secrete IL-2, interferon-γ (IFN-γ), tumor necrosis factor (TNF), and antagonistic cytokines of IgE. TH2 cytokines, such as IL-4, IL-5 and IL-13, induce IgE production and activate eosinophilia. AR treatment contributes to the downregulation of TH2 cytokines [35]. IL-1β is a significant pro-inflammatory cytokine produced by epithelial cells in nasal mucosa. Studies indicated that AR is a persistent inflammation that promotes eosinophil activation and IL-1β upregulation [36]. IL-1β is required for the induction of ovalbumin specific T cells and local inflammatory in the sensitization and elicitation phase [37]. Furthermore, IL-1β enables B cell to differentiate into plasma cells that secrete IgE, thereby enhancing mast cell activation mediated by TH2 cytokines, leading to IgE production (Fig. 5) and ultimately to the development of AR [38–40]. rhIL-1Ra, a natural regulator and antagonist of IL-1, binds the receptors to inhibit multiple activities of IL-1 without induction of downstream signaling cascades [41]. We speculate that rhIL-1Ra may suppress the production of IgE and finally block the release of inflammatory cytokines. Our results showed that IL-1β and ovalbumin specific IgE levels were markedly increased in ovalbumin-mediated AR guinea pig models (Fig. 3A and B). However, administration with rhIL-1Ra decreased IL-1β and ovalbumin specific IgE levels (Fig. 3A and B). Meanwhile, rhIL-1Ra significantly reduced the shedding of nasal mucosa epithelial cells, the infiltration of eosinophils, the tissue edema and the vasodilation comparing to the model group (Fig. 4A and B). These data further confirmed our speculation that rhIL-1Ra may decrease IgE levels and thus inhibit the release of inflammatory cytokines, like IL-1β.

In conclusion, in contrast to antihistamines and steroids, rhIL-1Ra has been approved by the FDA to bind specifically to IL-1R and inhibit IL-1-mediated multiple inflammatory diseases. We successfully constructed AR guinea pig models and found for the first time that rhIL-1Ra exhibited a strong therapeutic efficacy on guinea pigs with AR. The anti-AR effects were closely associated with reduction of IL-1β, and ovalbumin specific IgE levels and alleviation of epithelial cell exfoliation, eosinophilic infiltration, tissue edema and vascular dilatation (Fig. 5). This study provides additional options for the safe and effective treatment of AR.

## Conclusions

In conclusion, this study implies that rhIL-1Ra is effective in AR guinea pigs and may be a novel promising drug for AR.

## References

[1] Jin C, Ye K, Luan H, Liu L, Zhang R, Yang S (2020). Tussilagone inhibits allergic responses in OVA-induced allergic rhinitis guinea pigs and IgE-stimulated RBL-2H3 cells. Fitoterapia.

[2] Stahl SM (2008). Selective histamine H1 antagonism: novel hypnotic and pharmacologic actions challenge classical notions of antihistamines. CNS Spectr.

[3] Kalpaklioglu F, Baccioglu A (2012). Efficacy and safety of H1-antihistamines: an update. Antiinflamm Antiallergy Agents Med Chem.

[4] Waddell AN, Patel SK, Toma AG, Maw AR (2003). Intranasal steroid sprays in the treatment of rhinitis: is one better than another?. J Laryngol Otol.

[5] Hossenbaccus L, Linton S, Garvey S, Ellis AK (2020). Towards definitive management of allergic rhinitis: best use of new and established therapies. Allergy Asthma Clin Immunol.

[6] Ritvo PG, Klatzmann D (2019). Interleukin-1 in the Response of Follicular Helper and Follicular Regulatory T Cells. Front Immunol.

[7] Firestein GS, Boyle DL, Yu C, Paine MM, Whisenand TD, Zvaifler NJ (1994). Synovial interleukin-1 receptor antagonist and interleukin-1 balance in rheumatoid arthritis. Arthritis Rheum.

[8] Martin D, Near SL (1995). Protective effect of the interleukin-1 receptor antagonist (IL-1ra) on experimental allergic encephalomyelitis in rats. J Neuroimmunol.

[9] Wang X, Gao J, Qian L, Gao J, Zhu S, Wu M (2015). Exogenous IL-1Ra attenuates intestinal mucositis induced by oxaliplatin and 5-fluorouracil through suppression of p53-dependent apoptosis. Anticancer Drugs.

[10] Ter Haar NM, van Dijkhuizen EHP, Swart JF, van Royen-Kerkhof A, El Idrissi A, Leek AP (2019). Treatment to target using recombinant Interleukin-1 receptor antagonist as First-Line Monotherapy in New-Onset systemic juvenile idiopathic arthritis: results from a five-year Follow-Up study. Arthritis Rheumatol.

[11] Oelmann E, Topp M, Reufi B, Papadimitriou C, Koenigsmann M, Oberberg D (1994). Interleukin-1 receptor antagonist inhibits growth modulation of human tumor-cell lines by interleukin-1 in-vitro. Int J Oncol.

[12] Chen Y, Yang Z, Deng B, Wu D, Quan Y, Min Z (2020). Interleukin 1beta/1RA axis in colorectal cancer regulates tumor invasion, proliferation and apoptosis via autophagy. Oncol Rep.

[13] Zheng Y, Xiao X, Yang Z, Zhou M, Chen H, Yuan Y (2019). Protective effect of recombinant human IL-1Ra on hepatocytes in vitro. J Shanghai Jiaotong Univ (Medical Science).

[14] Underwood S, Foster M, Raeburn D, Bottoms S, Karlsson JA (1995). Time-course of antigen-induced airway inflammation in the guinea-pig and its relationship to airway hyperresponsiveness. Eur Respir J.

[15] Bahekar PC, Shah JH, Ayer UB, Mandhane SN, Thennati R (2008). Validation of guinea pig model of allergic rhinitis by oral and topical drugs. Int Immunopharmacol.

[16] Brozmanova M, Calkovsky V, Plevkova J, Bartos V, Plank L, Tatar M (2006). Early and late allergic phase related cough response in sensitized guinea pigs with experimental allergic rhinitis. Physiol Res.

[17] Xiao H, Xie Y, Li B, Li H, Di C, Hu M (2012). Establishment and characteristics of a TDI-induced model of allergic rhinitis in guinea pigs. Military Med J Southeast China.

[18] Al Suleimani M, Ying D, Walker MJ (2007). A comprehensive model of allergic rhinitis in guinea pigs. J Pharmacol Toxicol Methods.

[19] Zhang HQ, Sun Y, Xu F (2003). Therapeutic effects of interleukin-1 receptor antagonist on allergic rhinitis of guinea pig. Acta Pharmacol Sin.

[20] Tanaka K, Okamoto Y, Nagaya Y, Nishimura F, Takeoka A, Hanada S (1988). A nasal allergy model developed in the guinea pig by intranasal application of 2,4-toluene diisocyanate. Int Arch Allergy Appl Immunol.

[21] Yan Z, Liu L, Yuan J, Jiao L, Zhou M, Liu J (2021). Yiqi Jiemin decoction alleviates allergic rhinitis in a guinea pig model by suppressing inflammation, restoring Th1/Th2 balance, and improving cellular metabolism. Aging.

[22] Tang H, Li T, Han X, Sun J (2019). TLR4 antagonist ameliorates combined allergic rhinitis and asthma syndrome (CARAS) by reducing inflammatory monocytes infiltration in mice model. Int Immunopharmacol.

[23] Zhu YQ, Liao B, Liu YH, Wang Z, Zhu XH, Chen XB (2019). MicroRNA-155 plays critical effects on Th2 factors expression and allergic inflammatory response in type-2 innate lymphoid cells in allergic rhinitis. Eur Rev Med Pharmacol Sci.

[24] Tu W, Chen X, Wu Q, Ying X, He R, Lou X (2020). Acupoint application inhibits nerve growth factor and attenuates allergic inflammation in allergic rhinitis model rats. J Inflamm (Lond).

[25] Morjaria JB, Caruso M, Emma R, Russo C, Polosa R (2018). Treatment of allergic Rhinitis as a strategy for preventing asthma. Curr Allergy Asthma Rep.

[26] Kawauchi H, Yanai K, Wang DY, Itahashi K, Okubo K (2019). Antihistamines for allergic Rhinitis treatment from the viewpoint of Nonsedative Properties. Int J Mol Sci.

[27] Al Suleimani YM, Walker MJ (2007). Allergic rhinitis and its pharmacology. Pharmacol Ther.

[28] Christodoulopoulos P, Cameron L, Durham S, Hamid Q (2000). Molecular pathology of allergic disease. II: Upper airway disease. J Allergy Clin Immunol.

[29] He SH, Zhang HY, Zeng XN, Chen D, Yang PC (2013). Mast cells and basophils are essential for allergies: mechanisms of allergic inflammation and a proposed procedure for diagnosis. Acta Pharmacol Sin.

[30] Varricchi G, Rossi FW, Galdiero MR, Granata F, Criscuolo G, Spadaro G (2019). Physiological roles of mast cells: Collegium Internationale Allergologicum Update 2019. Int Arch Allergy Immunol.

[31] Wu YR, Hsing CH, Chiu CJ, Huang HY, Hsu YH (2022). Roles of IL-1 and IL-10 family cytokines in the progression of systemic lupus erythematosus: friends or foes?. IUBMB Life.

[32] Boraschi D, Italiani P, Weil S, Martin MU (2018). The family of the interleukin-1 receptors. Immunol Rev.

[33] Wang C (2013). Identification and testing of recombinant human interleukin-1 receptor antagonist.

[34] Benson M, Strannegard IL, Strannegard O, Wennergren G (2000). Topical steroid treatment of allergic rhinitis decreases nasal fluid TH2 cytokines, eosinophils, eosinophil cationic protein, and IgE but has no significant effect on IFN-gamma, IL-1beta, TNF-alpha, or neutrophils. J Allergy Clin Immunol.

[35] Yang HC, Won EJ, Kim MJ, Sung CM, Rhee JH, Nam KI (2021). Intralymphatic Administration of Metagonimus yokogawai-extracted protein attenuates experimental murine allergic Rhinitis Model. Int Arch Allergy Immunol.

[36] Kim HY, Nam SY, Jang JB, Choi Y, Kang IC, Kim HM (2016). 2-(4-2-[(phenylthio)acetyl]carbonohydrazonoylphenoxy)acetamide as a new lead compound for management of allergic rhinitis. Inflamm Res.

[37] Nambu A, Nakae S (2010). IL-1 and Allergy. Allergol Int.

[38] Galli SJ, Tsai M (2012). IgE and mast cells in allergic disease. Nat Med.

[39] Han MW, Kim SH, Oh I, Kim YH, Lee J (2019). Serum IL-1beta can be a biomarker in children with severe persistent allergic rhinitis. Allergy Asthma Clin Immunol.

[40] Shi Q, Lei Z, Cheng G, Li D, Wang Q, Luo S (2018). Mitochondrial ROS activate interleukin-1beta expression in allergic rhinitis. Oncol Lett.

[41] Hannum CH, Wilcox CJ, Arend WP, Joslin FG, Dripps DJ, Heimdal PL (1990). Interleukin-1 receptor antagonist activity of a human interleukin-1 inhibitor. Nature.

